# The Effect of Pre-Incision Field Block versus Post-Incision Inguinal Wound Infiltration on Postoperative Pain after Paediatric Herniotomy

**DOI:** 10.3889/oamjms.2015.116

**Published:** 2015-11-14

**Authors:** Simeon Olafimihan Olanipekun, Oyebola Olubodun Adekola, Ibironke Desalu, Olusola Temitayo Kushimo

**Affiliations:** 1 *Department of Anaesthesia & Intensive Care Unit, Lagos University Teaching Hospital, Lagos, Nigeria*; 2 *Department of Anaesthesia & Intensive Care Unit; College of Medicine University of Lagos & Lagos University Teaching Hospital, Lagos, Nigeria*

**Keywords:** Preincisional ilioinguinal/iliohypogastric nerve block, postincisional wound infiltration, pain score, Herniotomy

## Abstract

**BACKGROUND:**

The Ilioinguinal/iliohypogastric nerve block has been shown to significantly decrease opioid analgesic requirements and side effects after inguinal herniotomy. We compared the effect of pre-incisional field block with 0.25% bupivacaine and post-incisional wound infiltration with 0.25% bupivacaine for postoperative pain control after inguinal herniotomy.

**PATIENTS & METHODS:**

This was a randomized controlled double blind study in 62 ASA I and II children aged 1-7 years scheduled for inguinal herniotomy. They were assigned to receive either pre-incision field block (group I) or post-incision wound infiltration at the time of wound closure (group II). The pain score was assessed in the recovery room using mCHEOPS score and VAS or FLACC score at home by the parents for 24 hours.

**RESULTS:**

The mean pain scores during the 2 hour stay in the recovery room, at 12 and 18 hours at home were similar in both groups, p > 0.05. However, the mean pain scores were significantly lower at 6 hours at home in group I (1.22 ± 0.57) than in group II (1.58 ±0.90), p <0.001, but significantly higher at 24 hours at home in group I (3.29 ± 0.46) than in group II (2.32 ± 0.24), p = 0.040. There was no difference in mean paracetamol requirement, and in the number of patients who required paracetamol for pain relief at home in both groups, p > 0.05.

**CONCLUSION:**

We have demonstrated that both pre-incisional ilioinguinal/iliohypogastric field block and post incisional wound infiltration provided adequate postoperative analgesia for 24 hours after inguinal herniotomy.

## Introduction

Ilioinguinal/iliohypogastric field block appears to be the technique of choice in many paediatric institutions in Europe and America because it is usually effective with minimal risk, and low cost [1]. The supplementation of analgesia with either a non-steroidal anti-inflammatory drug or caudal block has been reported to further improve analgesia [2]. Acute postoperative pain is one of the most adverse stimuli experienced by children. Pain is, however, often under treated in the paediatric age group [3, 4]. This is attributable to barriers to pain control in children, such as the myths that children and infants do not feel pain, they do not remember painful episodes, and that there is no untoward consequence of experiencing pain [3]. Eske et al. demonstrated that 12.4% of paediatric patients were found to have persistent post herniotomy pain [4].

Uncontrolled pain can induce tachycardia, raised blood pressure, insomnia and behavioural disorders [5]. In view of these, there is a need to investigate how pain can be prevented before its onset. The concept of pre-emptive analgesia is based on treating the pain before it is provoked, by preventing the pain signals from reaching the spinal cord or brain [6]. This is achieved by anticipating the mechanism of its causation and preventing peripheral and central sensitization with carefully chosen therapy [7, 8].

The use of pre-emptive analgesia may involve pre-incisional nerve blocks, injection of local anaesthetic agents to the site of the incision or regional blocks like caudal block. This study investigated the effectiveness of pre-emptive analgesia using pre-incisional field block for herniotomy with 0.25% plain bupivacaine compared with post incisional wound infiltration with 0.25% plain bupivacaine on the pain scores in paediatrics patients in the immediate postoperative period.

## Patients and Methods

This was a prospective, double blind randomised controlled trial in 62 American Society of Anesthesiologist (ASA) physical status I or II subjects aged 1-7 years scheduled for ambulatory inguinal herniotomy. The institution Human Research Ethics Committee approval and informed consent were obtained from subject’s parents/relatives. The recovery room nurse was educated on the use of the modified Children’s Hospital of Eastern Ontario Pain Scale (mCHEOPS) [9]. The parents were also educated on the use of the visual analogue scale (VAS) for older children > 5 years. In this, the child was to indicate on a 10cm line marked with one end (0 cm) representing no pain and the other (10 cm) representing the worst pain. The Face, Legs, Activity, Cry, Consola-bility (FLACC) scale was used for children < 5 years. They were informed that they would be contacted the day after surgery via a phone call to assess the presence of pain.

The patients were randomly allocated by blind balloting to one of the two groups by using a sealed envelope technique. Group I received 1 ml/kg of 0.25% bupivacaine 20 minutes before surgical incision, and equal volume of normal saline infiltration at skin closure. Group II received 1 ml/kg of normal saline 20 minutes before surgical incision and 1 ml/kg of 0.25% bupivacaine at skin closure. The surgeon who was blind to the type of solution in the syringes performed the field block.

Patients with sickle cell disease, liver, renal, respiratory or cardiovascular diseases, those on chronic analgesic therapy, ASA III and IV and BMI > 85 percentile for age and sex were excluded from the study.

### Anaesthetic procedure

Routine electrolytes/urea/creatinine, haemoglobin electrophoresis and packed cell volume were done at the surgical outpatient unit at least one week before surgery. Preoperative assessment was conducted on the morning of surgery; fasting guideline was 6 hours to solid food, 4 hours to breast milk and 2 hours to clear fluid. No premedication was administered. The weight and height were taken on the morning of surgery.

On arrival in the theatre, multiparameters monitor (Datex Ohmeda Cardiocap 7100) Metropolitan Medical Services of NC. Inc. 15 Westside Drive, Asheville North Carolina] was attached. Intraoperative monitoring included precordial stethoscope, non-invasive blood pressure, and electrocardiogram (ECG), heart rate and oxygen saturation. End tidal carbon dioxide (ETCO_2_) was measured after insertion of laryngeal mask airway (LMA).

All patients were induced with halothane (1-4.0%) incremental doses in 100% oxygen via Mapleson D Bains Circuit) for patients > 25 kg or Mapleson F breathing circuit (Jackson Rees modification of the Arye T piece), Flexicare, Mid Glamorgan; CF45 4ER UK, for patients < 25kg weight. When anaesthesia was considered adequate, intravenous (IV) access was secured with a 20-22 gauge cannula, and the airway was secured with an appropriate sized LMA.

The first infiltration was done after confirming adequacy of ventilation. The attending anaesthetist was responsible for preparing and labelling the solutions for infiltration. The surgeon was blinded to the type of solution in the syringe.

The field block was performed using a 21G hypodermic needle, the local anaesthetic agent was divided into four equal volumes of 5 ml each. Four sites were infiltrated viz 1-2 cm medial to the anterior superior iliac spine and to the external oblique aponeurosis, 1.5 cm above the midpoint of inguinal ligaments, the subcutaneous tissue from the pubic tubercle towards the umbilicus and the subcutaneous tissue along the line of the skin incision.

Anaesthesia was maintained with Isoflurane (1-2.5%) in 100% oxygen which was adjusted to achieve stable intraoperative haemodynamic measurements. All patients received intravenous paracetamol 15 mg/kg and diclofenac 1 mg/kg 20 minutes after the field block was performed before surgical incision. The administration of intravenous fluids in the operating room followed standard guidelines for paediatric patients.

At the end of surgery, the second infiltration was performed by the same surgeon. Anaesthesia was discontinued and the LMA was removed in all patients when they were fully awake. The children were continuously monitored in the recovery room every 15 minutes for 2 hours, after which they were discharged home if they were fit for discharge. During this time, a nurse blinded to the two groups assessed the pain score using mCHEOPS on arrival in the recovery room at 0 minute and at 15, 30, 45, 60, and 120 minutes post-operatively. A score of < 6 was taken to be satisfactory pain control. A score of > 6 was taken as moderate to severe pain and IV pentazocine 0.5 mg/kg was given as the supplemental analgesia. The time of first analgesic requirement was noted. Parents were instructed on how to assess pain using VAS or FLACC scales at home every six hours for 24 hours after discharge. Oral paracetamol 15 mg/kg was given at home for severe pain (> 6) in ages 4-7 years and those between ages 1-3 years who refused food and could not be consoled following crying. The frequency of use of additional paracetamol, and the total analgesic used at home was obtained by the researcher via a phone call.

*The following data were recorded:* Pain scores assessed at 0, 15, 30, 45, 60 and 120 minutes in the recovery room. The incidence of vomiting, the use and timing of supplemental analgesic (pentazocine) in the recovery room, the time to first micturition and the time to first analgesic requirement.

Parents were contacted the day after surgery and asked to report the following: The pain score since discharge from the hospital every 6 hours, the occurrence of vomiting, the time to first micturition and total amount of additional paracetamol given at home. The primary outcome compared the effect of pre-emptive analgesia of pre-incisional field block using 0.25% bupivacaine for postoperative pain control with postincisional infiltration of 0.25% bupivacaine at the time of wound closure, in patients aged 1-7 year old scheduled for elective unilateral herniotomy. The secondary outcome determined the pain scores, the time to first administration of analgesia and the frequency of administration of oral paracetamol in patients scheduled for herniotomy with pre-incisional field block or post-incisional infiltration of 0.25% bupivacaine wound infiltration. We tested the alternate hypothesis, that the pain relief obtained with pre-incisional ilioinguinal/iliohypogastric field block using 0.25% bupivacaine was significantly superior to that obtained with post-incisional wound infiltration using 0.25% bupivacaine in children scheduled for unilateral inguinal herniotomy.

### Statistical analysis

Data collection was done by the researcher who was blinded to the groups. Data were presented as mean ±SD, frequencies, and percentile, and the differences between the two groups were analysed using the student t-test for or chi-square as considered appropriate, p ≤ 0.05 was considered significant. All analysis was performed using the Statistical Package for Social Sciences for Windows version 17 (SPSS, Chicago, IL).

## Results

Sixty-two patients, (31 in each group) were recruited for the study. There was no significant difference in the mean age, weight, height, BMI duration of surgery, the time of first analgesic requirement, total dose of paracetamol consumed, and the time of first micturition between the groups (Table 1). There was no pain on arrival at the recovery room and during the 2 hours stay in both groups; therefore no patients in either group received supplemental analgesia (pentazocine) during their recovery room admission.

**Table 1:** The Demographic and Clinical Characteristics of Patients

Variable	Group I (Mean ± SD) n = 31	Group II (Mean ± SD) n = 31	p value
Age (years)	3.50 ± 1.71	3.06 ± 1.94	0.687
Weight (kg)	15.26 ± 4.34	13.74 ± 4.53	0.800
Height (m)	0.96 ± 0.14	0.91 ± 0.14	0.667
BMI (kg/m^2^)	16.20 ± 1.10	16.39 ± 1.45	0.588
Duration of surgery (minutes)	32.97 ± 4.59	23.22 ± 2.36	0.071
Time of first analgesic requirement (hours)	13.12 ± 6.57	11.37 ± 6.74	0.333
Total dose of PCM (mg ± SEM)	316.07 ± 99.15	318.52 ± 115.28	0.479

Data represents mean ±SD, and p value for age, weight, height, BMI, Duration of surgery, Time of first rescue analgesia, Total dose of PCM given.

The distribution of pain within 24 hours of surgery and the mean pain scores at rest after surgery in the two groups are shown in Tables 2 and 3. The mean pain score was significantly lower at 6 hours in group I (1.22 ± 0.58) than group II (1.58 ± 0.90), p <0.001, but higher at 24 hours; in group I (3.29 ± 0.46) than group II (2.32 ± 0.24), p = 0.040. However, the mean pain scores were similar at 12 and 18 hours during the study period, p > 0.05.

**Table 2:** The effect of pre-incisional field block versus post-incisional wound infiltration on number of patients with pain at home

Time at home	Group I No of patient remaining in study		Group II No of patient remaining in study		df	p value

	n	f	n	f		
Arrival at home	31	1 (3.23%)	31	0 (0%)	4	0.1
6 hours	30	8 (26.67%	31	4 (12.9%)	4	0.001
12 hours	22	8 (30.77%)	27	11 (47.83%)	4	0.005
18 hours	14	6 (28.57%)	16	5 (29.41%)	4	0.28
24 hours	8	4 (23.53%)	11	4 (30.77%)	4	0.051

Data represent the number of patients which had pain during the 24 hour period. Values are frequency, degree of freedom and chi-square.

**Table 3:** The effect of Pre-incisional field block and Post-incisional wound infiltration on Pain Score at home

Variable	Group I Mean ± SD	Group II Mean ± SD	df	p value
Pain score on arrival at home	1.00 ± 0.00	1.04 ± 0.44	60	0.072
Pain score at 6hr	1.22 ± 0.58	1.58 ± 0.90	51	< 0.001
Pain score at 12hrs	1.83 ± 0.94	1.78 ± 0.94	39	0.871
Pain score at 18hrs	1.62 ± 0.11	1.64 ± 0.15	21	0.588
Pain scores at 24hrs	3.29 ± 0.46	2.32 ± 0.24	10	0.040

**Figure 1:**
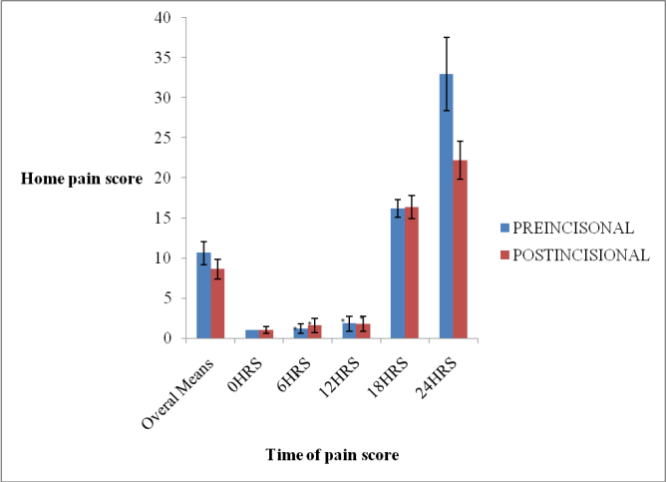
The effect of Pre-Incisional Illio-Inguinal block and Post-incisional wound infiltration on pain score at home. This figure shows the mean pain score at home. Values are mean ± SD. *Indicates that the difference between was significant at p < 0.05.

The mean paracetamol consumption at home was similar in both groups; group I (316.07 ± 99.15) mg versus group II (318.52 ± 115.28) mg, p = 0.497. However, the number of patients who required paracetamol in group II (24) was less than in group I (27) though this was not significantly different, p = 0.167.

There were significant differences in the number of patients who had pain between the groups at 6 and 12 hours, (p <0.05), Table 2. The subjects without pain were exempted from the next assessment as delineated in Table 2. Only two patients in group II developed bronchospasm.

## Discussion

We have demonstrated that either pre-incisional ilioinguinal/iliohypogastric nerve block or post-incisional wound infiltration using 0.25% bupivacaine were effective in relieving postoperative inguinal herniotomy pain in children. There was no significant difference in mean pain scores in the first 2 hour (mean score of both were zero) period of admission in the recovery room. We observed that the mean pain scores at home in both groups were <6, which was taken to be a reflection of satisfactory pain control. This may suggest that both techniques of analgesia were effective in the control of postoperative herniotomy pain. This is agreement with observation made by Sajedi et al. [10] which we attributed to the similarity in techniques of nerve block and wound infiltration. However, the difference in level of significance in pain score between the studied groups did not follow a consistent pattern during the study period. At 6 hours postoperatively, the mean pain score was significantly lower in the pre-incisional field block group than in the post-incisional wound infiltration group. While at 24 hours, the reverse was the observed. Our observation at 24 hours, we attributed to the initial delay of 20 minutes in the pre-incisional group after the block before incision, which would have contributed to a prolongation in the time of assessment beyond 24 hours in the pre-incisional group. This is a possible limitation to this study, in future studies we suggest that the time for pain assessment should be commenced from wound infiltration or incision time and not at the end of surgery was done by us.

In contrast, Sajedi et al. [10] reported that the mean pain scores were significantly lower at recovery, 6, 12, and 24 hours after surgery in the pre-incisional field block group than in the post-incisional wound infiltration group. The variability in the two studies, we attributed to the fact that the ilioinguinal/iliohypogastric field block was performed by different surgeons in our study. Unlike, the later where the same surgeon performed the field block and wound infiltration [10]. This might have reduced the effect of operator skill on the performance of the nerve block, adequacy of block and its success rate. The technique and tool for pain assessment may also be contributory. In our study, herniotomy was done as an ambulatory surgery and patients were discharged after 2 hours of observation. The recovery nurses evaluated the pain score using mCHEOPS in the recovery room, while VAS or FLACC scale was used by the parents at home. In contrast, a dedicated observer evaluated the pain score with Oucher index score in the Sajedi’s study [10]. Nevertheless, it has been previously documented that the assessment of the need for analgesia by attending nursing staff varies widely and is unreliable [11].

The mCHEOPS appears to be more complex and therefore the interpretation may be guarded, unlike the Oucher pain scale that uses a numerical scale or facial expression index.

On arrival in the recovery room, none of the patients in our study experienced pain using the mCHEOPS score (score of 0) during their two hour stay. In contrast, Reid et al. [12] observed that with the use of linear analogue score, 56% of the patients in the pre-incisional field block group were pain free on arrival in the recovery room, of which 68% had a score less than two. In those who received wound infiltration, 100% were pain free, and 86% of the patients had a score < 2. The difference in the reported incidence of pain may be due to the difference in the pain assessment tools and the competence of the assessor.

The number of subjects who complained of pain in our study was significantly lower in the pre-incisional field block group than the post-incisional group. Reid et al. [12] in a similar population of patients observed that the use of ilioinguinal nerve blockade for postoperative analgesia in children was associated with increase in the number of pain free patients, and a significant reduction in postoperative analgesia requirements. The use of the number of patients to monitor the control of postoperative pain after herniotomy may not be an objective method of monitoring pain especially in children. Other researchers had earlier suggested that the mean pain score and the time to first analgesic requirement were better indicators of adequacy of pain relief [12].

In contrast to our observation, Saejdi et al. [10] reported a significant reduction in analgesic consumption in the pre-incisional field block group. Saejdi concluded that pre-incisional field block was a useful and better method of alleviating postoperative pain than post-incisional wound infiltration during herniotomy. This observation is surprising because of the similarity in the time of injection of bupivacaine during pre-incisional field block and post incisional wound infiltration in both studies. The pre-incisional field block was established 20 minutes before skin incision, the four point injection technique of local anesthetic agent for the field block, and the same dose of bupivacaine was used (0.25%). However, the use of supplemental paracetamol and diclofenac may be responsible), as Saejdi [10] did not use additional analgesics. This might have influenced the requirement for additional analgesia. In contrast, Langer et al. [13] reported that the pre-emptive use of bupivacaine resulted in lower analgesic requirements in the immediate postoperative period and at home, allowing earlier ambulation than the control (saline) group.

Similarly, Dahl et al. [14] observed that pre-emptive pre-incisional bupivacaine significantly reduced the halothane requirement and objective pain score at 30 minutes during herniotomy when compared with the post-incisional saline. However, there were no differences between the two groups regarding the need for additional analgesia; even though pre-incisional field block was shown to have sparing effects on meperidine [14]. Other methods of pain relief for postoperative herniotomy include caudal block, which has been reported to have the same efficacy with ilioinguinal/iliohypogastric field block [15].

The mean time to first postoperative analgesic requirement in this study is similar to that reported with postincisional wound infiltration after herniotomy [16]. Unlike our study, similar studies on pre-emptive analgesia compared the ilioinguinal/iliohypogastric field block or postincisional wound infiltration with placebo [13, 16]. This is because the leading ethical position on placebo controlled clinical trials is that whenever proven effective treatment exists for a given condition, it is unethical to test a new treatment for that condition against placebo. Nevertheless, placebo controlled studies are ethically justifiable when they are supported by sound methodological considerations, and their use does not expose research participants to excessive risks of harm [17].

The mean time of micturition in our study was similar in both groups; unlike observations made with caudal block by Edomwonyi et al. [15] when they reported that the time to micturition was longer in the caudal analgesia group than in the pre-incisional field block group.

None of our cohort experienced fever and wound dehiscence, a similar observation was made by like Reid et al. [12] the observation, they related to the antimicrobial activity of bupivacaine, which may protect against wound infection.

The use of ultrasound guided ilioinguinal block has been reported to provide a better quality of analgesia than the standard “fascial click” method [3]. In addition, it reduced the volume of local anaesthetic thereby decreasing the risk of toxicity and untoward side effects [17]. The National Institute for Health and Clinical Excellence (NICE) guidelines recommend the use of a 2-D imaging ultrasound guidance as a preferred method for peripheral nerve block, and regional anaesthesia in adults and children [19].

The study has some limitations, in the pre-incisional nerve block group, there was no ultrasound available in our institution during the study period. An ultrasound guided nerve localisation technique will ensure proper identification and blockade of the ilioinguinal/iliohypogastric nerves. The nerve block and local infiltration was done by different surgeons with varied experience, this may constitute bias and increase the chance of a failed block. Similarly, variation in pain assessment by parents at home might have influenced the pain score assessment, future studies using domiciliary nurses will help to eliminate the bias.

We have demonstrated that either pre-incisional ilioinguinal/iliohypogastric nerve block or post-incisional wound infiltration with bupivacaine (0.25%) was effective for postoperative pain management following paediatric herniotomy. These techniques are very simple, cost effective with minimal time taking. This seems to be the best method of pain management in children for day case surgery. When a child is playful; the parents are also satisfied physically as well as psychologically.
